# Supplementary material for: Multimodal imaging and functional analysis of the chick NMDA retinal damage model
Source: PLoS One. 2021 Sep 7;16(9):e0257148. doi: 10.1371/journal.pone.0257148 (PMC8423281; doi:10.1371/journal.pone.0257148)
Supplement: S1 File — (ZIP) [file pone.0257148.s004.zip › SD-OCT/C200727/011/D15-PWk3/C200727-011_OD_V_14X14_0_REGAVG0000064/Manual_PreDefineFiveByFiveGridRectangularRetina_1.pdf]

Five By Five Grid Template Report

**Patient ID**	C200727	**Name**	See InVivoVue
**Exam Date**	See InVivoVue	**Exam Time**	See InVivoVue
**Eye**	OD	**Averaged**	N/A
**Depth (mm)**	1.65	**Depth Samples**	1024
**Pitch (um/pix)**	35.000 x 35.000 x 1.608	**Refractive Index**	See InVivoVue
**Scan Type**	Rectangular 14.000mm x 14.000mm x 1.647mm , 400 x 400 x 1024		

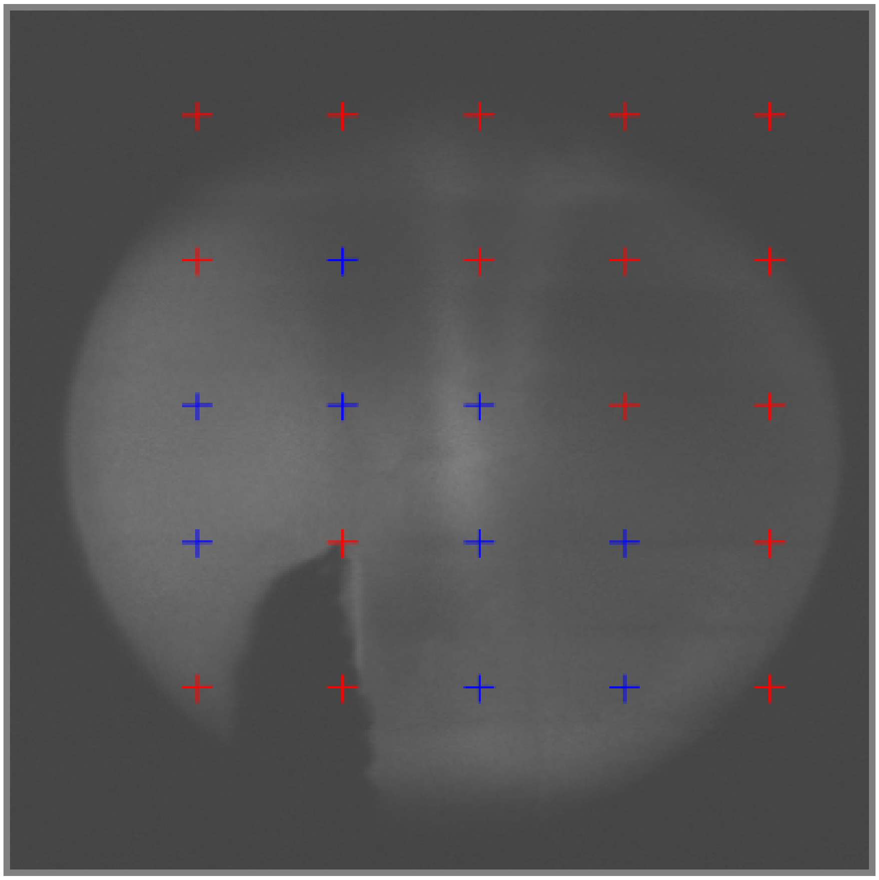

Thickness Metrics

Analysis	Average(mm)	Minimum(mm)	Maximum(mm)	STDEV(mm)
**IRNFL - RPE**	0.2690	0.2540	0.2808	0.0083
**IRNFL - ORNFL**	0.0418	0.0225	0.0605	0.0150
**ORNFL - OPL**	0.1536	0.1367	0.1772	0.0163
**OPL - ETPRS**	0.0563	0.0530	0.0630	0.0033

Q:\Bioptigen\Data\images\site3000254\image\2020.08.17\C200727-011_OD_V_14X14_0_REGAVG0000064.OCT

1/6/2021 3:40:34 PM
